# Esophageal Capsule Endoscopy for Screening Esophageal Varices among Japanese Patients with Liver Cirrhosis

**DOI:** 10.1155/2012/946169

**Published:** 2011-11-30

**Authors:** Haruya Ishiguro, Shoichi Saito, Hiroo Imazu, Hiroyuki Aihara, Tomohiro Kato, Hisao Tajiri

**Affiliations:** ^1^Division of Gastroenterology and Hepatology, Department of Internal Medicine, Jikei University School of Medicine, 3-25-8 Nishi-shimbashi, Minato-ku, Tokyo 105-8461, Japan; ^2^Department of Endoscopy, Jikei University School of Medicine, 3-25-8 Nishi-shimbashi, Minato-ku, Tokyo 105-8461, Japan

## Abstract

*Purpose*. Although esophageal capsule endoscopy (ECE) is reportedly useful in the diagnosis of esophageal varices (EV), few reports have described the benefits of this technique in Asian countries. The present paper evaluates the usefulness of ECE for diagnosing EV in Japanese patients with cirrhosis. *Methods*. We examined 29 patients with cirrhosis (20 males and 9 females; mean age 60 years; Child-Pugh classification A/B/C; 14/14/1) using ECE followed by esophagogastroduodenoscopy (EGD). High-risk EV were defined as F2 and/or RC2 and above. *Results*. The sensitivity and specificity of ECE for the diagnosis of high-risk EV were 92% and 80%, respectively. *Conclusions*. The findings showed that ECE is a highly sensitive method of diagnosing high-risk EV that requires endoscopic or pharmacological therapy. Thus, ECE might be a useful method for the screening and followup of EV in patients with cirrhosis.

## 1. Introduction

Esophageal varices (EV) arise when the hepatic-venous pressure gradient reaches >10–12 mmHg [1], and they comprise a serious complication of portal hypertension. The reported incidence of EV ranges between 5% and 12% [2], but about 90% of patients with cirrhosis will develop EV. The estimated annual rate of conversion of small EV to large EV in such patients is 12% [3]. Furthermore, between 25% and 30% of EV in cirrhotic patients will bleed. Despite advances in the management of acute variceal bleeding [4, 5], mortality rates after bleeding from EV remain at around 20% at 6 weeks. Primary prophylaxis with pharmacological agents or endoscopic treatment has been adopted to reduce mortality associated with variceal bleeding [6–8]. However, treatment with beta blockers and/or endoscopic band ligation is useful for the primary prophylaxis of variceal bleeding only in some patients with high-risk EV [1, 8–10]. Therefore, endoscopic screening is required to detect high-risk EV in patients with liver cirrhosis so that appropriate prophylactic treatment can be initiated [8, 10].

Esophageal capsule endoscopy (ECE) diagnostic systems (PillCam ESO; Given Imaging, Yokneam, Israel) enable visualization within the digestive tract including the esophagus, without the need for the administration of sedatives. If proven feasible and accurate for diagnosing EV in liver cirrhosis, this system could become an alternative method to standard upper gastrointestinal endoscopy.

Several reports have indicated that ECE can provide clear images of esophageal diseases, including Barrett's esophagus and esophagitis [11–14], and that it is useful for detecting EV [5, 13, 15]. However, few such reports have been generated from Asian countries, including Japan. Thus, the present prospective study assessed the feasibility and accuracy of ECE in diagnosing EV in Japanese patients with liver cirrhosis.

## 2. Patients and Methods

The present study compares the diagnostic accuracy of ECE for detecting EV compared with that of conventional esophagogastroduodenoscopy (EGD), which is the current gold standard for the EV diagnosis. Esophageal varices were recorded according to the general rules of the Japanese Society for Portal Hypertension [16] (Table 1). Endoscopic signs predictive of EV bleeding comprised moderate or large (F2 or F3) blue varices with marked red signs (RC2 or RC3) on their surface [17, 18]. According to the retrospective calculations of Imazu et al., these signs correspond to a bleeding risk of ≥64% [19]. Therefore, we defined high-risk varices as F2 and/or RC2 and above. The EGD reference and ECE findings were compared.

All of the enrolled patients had cirrhosis and some had suspected EV. The inclusion criteria were aged ≥18 years, prior endoscopic confirmation of EV and currently under clinical surveillance, or suspected portal hypertension with current endoscopic screening for EV. The exclusion criteria comprised a history of, or current dysphagia, known esophageal diverticulum, known or suspected intestinal obstruction, pregnancy, a history of gastrointestinal surgery other than uncomplicated cholecystectomy or appendectomy, having an implanted cardiac pacemaker or any other electromedical device and any condition that might preclude compliance with the study and/or the PillCam ESO instructions. The study proceeded in accordance with the ethical principles outlined in the Declaration of Helsinki, in compliance with good clinical practice and according to the Jikei University School of Medicine's regulations. The study protocol was approved by the Ethics committees of the Jikei University School of Medicine for biomedical research and institutional review boards. Written informed consent was obtained from all patients who met the inclusion criteria and agreed to participate in the study.

The patients fasted for at least 8 hours before undergoing ECE and EGD using a modification of the simplified ingestion protocol (SIP) [20]. Briefly, because EGD was started immediately after the patients had swallowed the ECE, pronase (proteases) was administered and then a data recorder including a recorder belt and sensor array was then attached to the patients. When the system was secured in place, seated patients ingested up to 50 mL of a mixture of water and liquid dimethicone (dimethylpolysiloxane) to reduce bubbles inside the esophagus. The patients then lay in the right lateral decubitus position and sipped water (approximately 15 mL per sip) before swallowing the ECE. The patient remained in the same position for 7 min and sipped water every minute. Pharyngeal anesthesia was applied to supine patients before being returned to the right lateral decubitus position for EGD. The ECE was the removed using a basket clamp where possible.

All findings generated using the ECE were reviewed by three experienced endoscopists who were blinded to each patient's history, with the exception of liver cirrhosis. All findings were documented as digital thumbnails. The examiner also documented the times of the first esophageal and gastric images so that the esophageal transit time could be determined. The examiner assessed the video for the presence or absence of EV and any EV present were graded using the Japanese endoscopic classification system [16].

Accuracy was assessed for the form of EVs detected and the presence of red signs. Rates of agreement between ECE and EGD, as well as the sensitivity and specificity of ECE, were determined.

## 3. Results

(1) Patients' backgrounds are shown in (Table 2). All 29 of the enrolled patients with cirrhosis (mean age, 66.0 ± 8.7 years; male, *n* = 20; female, *n* = 9) could ingest the capsule without difficulty, and no technical problems developed. None of the capsules were retained in the esophagus. Ten of the patients were under surveillance for EV, whereas the remaining 19 were undergoing screening. Nine patients had undergone previous endoscopic sclerotherapy. The median ECE transit time was 256.1 s (range, 4–917 s). The EGD findings indicated that of 22 patients with EV, 14, 14, and one had Child-Pugh classifications of A, B, and C, respectively. The ECE and EGD findings from 28 patients were assessed because the ECE did not reach the esophagogastric junction in one of them.

(2) Comparison of the accuracy of ECE and EGD in grading forms (F) of esophagogastric varices. The rates of agreement between the two techniques for F0, F1, F2, and F3 were 83%, 80%, 67%, and 66%, respectively.

(3) Comparison of the accuracy of the grading of red (RC) signs in esophagogastric varices by ECE and EGD.

The rates of agreement between the two techniques for RC0, RC1, RC2, and RC3 were 90%, 33%, 27%, and 0%, respectively.

**Figure 1:**
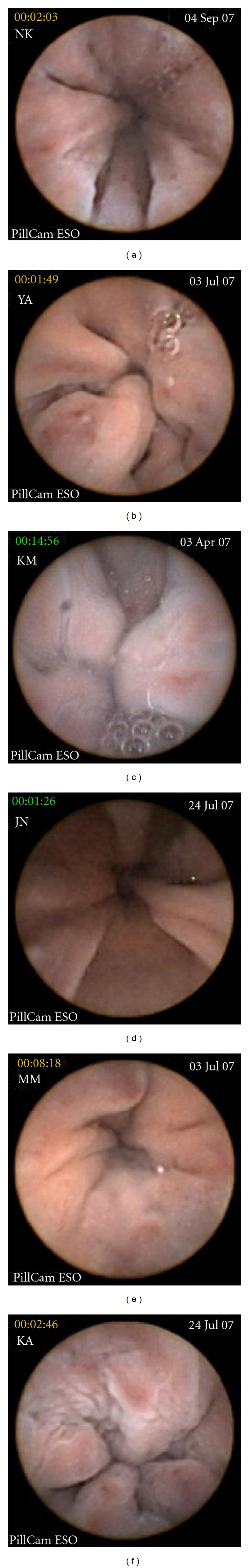
Images acquired using esophageal capsule endoscopy show (a–c) form (F) and (d–f) red (RC) signs of esophagogastric varices (EVs). Examples of EVs classified as F1 (a), F2 (b), and F3 (c), as well as RC0 (d), RC1 (e), and RC2 (f).

**Figure 2:**
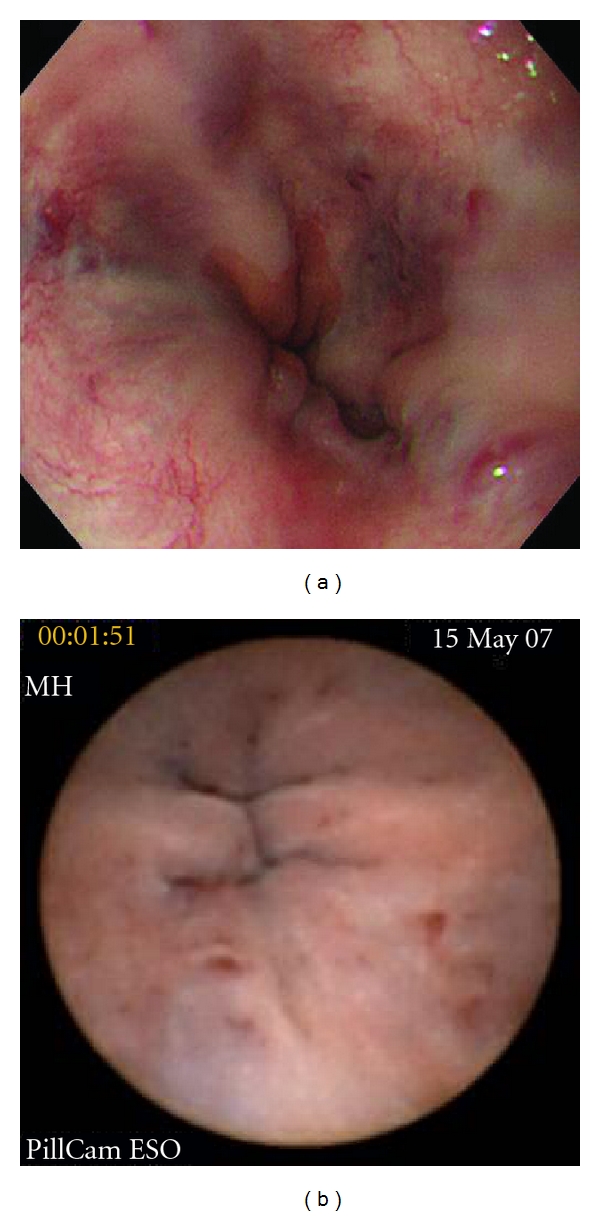
Images from a patient with good agreement between EGD (a) and ECE (b). This patient was classified as F1RC2 using both techniques.

**Figure 3:**
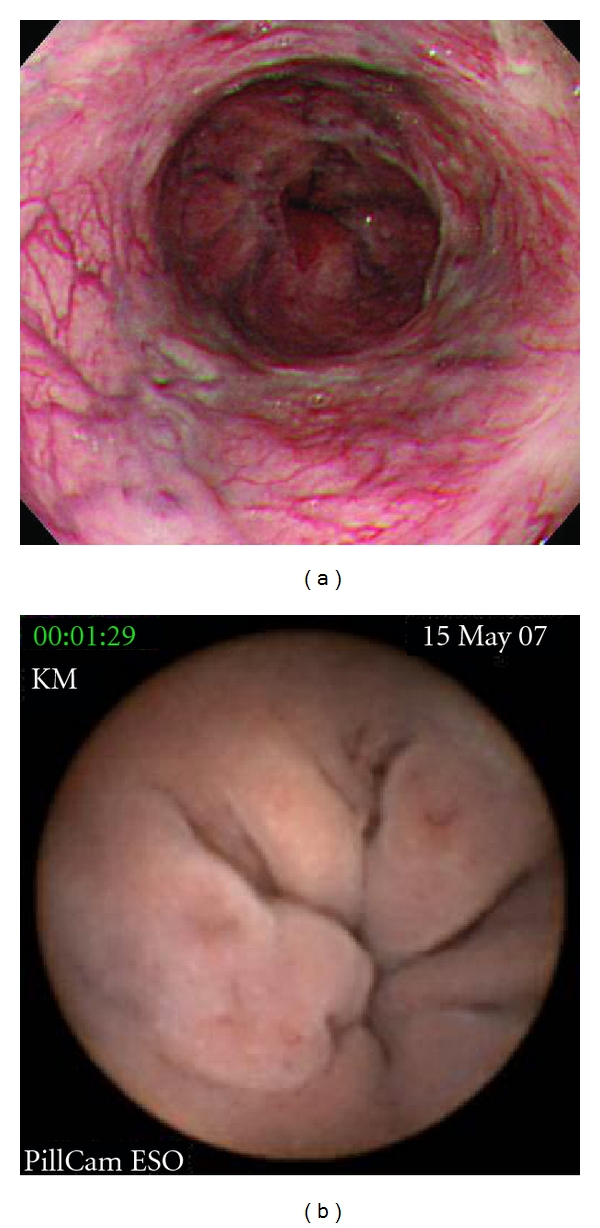
Images from a patient with poor agreement between EGD (a) and ECE (b). Varices were classified as F3RC1 by EGD (a), but as F2RC2 by ECE (b).

(4) Sensitivity and specificity of ECE for diagnosing EV (Table 3 and Figures 1–3). The sensitivity and specificity of ECE for diagnosing EV were 95% and 83%, respectively, whereas those of ECE for diagnosing RC on the variceal surface were 94% and 82%, respectively. Finally, the sensitivity and specificity of ECE for diagnosing high-risk EV requiring endoscopic therapy were 92% and 80%, respectively.

Figures 2 and 3 show images from patient in whom results obtained with ECE and EGD agreed and conflicted, respectively.

## 4. Discussion

Esophageal varices comprise one of the most serious complications of portal hypertension. Merli et al. [3] reported a 5% and 15% risk of hemorrhage from small and large EV, respectively. Two-thirds of bleeding EV will rebleed at some point, with a cumulative mortality of 33% [3]. Both the American Association for the Study of Liver Diseases (AASLD) and European liver societies recommend endoscopic screening for EV in patients with chronic liver diseases such as cirrhosis [21, 22].

Some reports have documented the application of ECE to screening for EV. For example, the pilot study of 32 patients by Eisen et al. found that the sensitivity and specificity ECE compared with EGD for evaluating EV was 100% and 89%, respectively [5]. Furthermore, Lapalus et al. found that ECE detected grade 2 EV and/or red signs in all of 21 patients that required primary prophylaxis [15] and Ramirez et al. reported that the accuracy of their string ECE for diagnosing EV and portal hypertension in patients with chronic liver disease was excellent [23]. In another study of 20 patients with cirrhosis who underwent ECE followed by EGD, the sensitivity of ECE for detecting EV was 68%, and ECE identified nine of 10 EV that had been rated grade 2 or higher on EGD [24]. Thus, the discrepancy between EGD and ECE appears greater when detecting smaller varices. Among 50 patients who underwent both ECE and EGD, the accuracy of ECE to determine the need for prophylaxis was 74%, with a sensitivity of 63% and a specificity of 82% [25]. A recent multicenter study of 288 patients found that the overall agreement between EGD and ECE for detecting EV was 85.8% and that the sensitivity and specificity of ECE was 84% and 88%, respectively [26]. In that study, the difference in the diagnosis of EV between the two techniques was 15.6% in favor of EGD. Finally, a French multicenter study of 120 patients found that the sensitivity and specificity of ECE for the indication of primary prophylaxis of 77% and 88%, respectively [27].

The sensitivity and specificity of ECE for a diagnosis of EV were 95% and 83%, respectively, compared with 94% and 82%, respectively, for the diagnosis of RC signs on the variceal surface. The sensitivity and specificity of ECE for the diagnosis of high-risk EV were 92% and 80%, respectively. Therefore, ECE can sensitively discriminate high-risk EV that require pharmacological or endoscopic therapy. Furthermore, ECE might be useful for the screening and followup of EV in patients with cirrhosis.

We used a modified SIP. Although the transit time was only 4 seconds in one of the patients, EVs could still be thoroughly examined because 56 images of the esophagus were acquired. Therefore, difficulties with analyzing ECE images are most often associated with esophageal secretions rather than with a short transit time. Eisen et al. reported that a major limitation of using ECE to grade EV was related to the lack of air insufflation [5]. However, given that grading is usually overestimated when using ECE compared with EGD, we believe that ECE can be used to grade EV.

We found a tendency to overestimate RC signs; in particular, RC1 was diagnosed as RC2. However, the findings around the esophagogastric junction taken by the ECE are probably true considering the clarity of the images, unlike those acquired using EGD.

Delvaux et al. examined 98 patients using ECE, but the capsule did not enter the stomach of 15 patients during a 20-minute recording period [28]. The capsule did not reach the esophagogastric junction in only one patient in the present study and was probably trapped in her stomach because the patient was lying down during both the ECE and EGD procedures.

A newer ECE model, namely, the PillCam ESO II (Given Imaging, Yokneam, Israel), has been developed that is supposed to acquire clearer images than the previous version. However, the data from the present study suggest that the PillCam ESO is sufficient for detecting EV. Further studies should determine differences in images obtained by the two ECE systems.

## Figures and Tables

**Table 1:** General rules for recording endoscopic findings of esophagogastric varices, as published by the Japanese Society for Portal Hypertension (2004) [16].

Form (F) Shape and size
F0: lesions assuming no varicose appearance
F1: straight small-calibered varices
F2: moderately enlarged, beady varices
F3: markedly enlarged, nodular, or tumor-shaped Varices)

Red color sign (RC)
Red wale marking, cherry Red Spot, hematocytic spot
RC0: absent
RC1: small in number and localized
RC2: intermediate between 1 and 3
RC3: large in number and circumferential

**Table 2:** Patients' background. ALLC, alcoholic liver cirrhosis; CLC, HCV-related liver cirrhosis; HCC, hepatocellular carcinoma; LC, liver cirrhosis NBNC, non-B, non-C liver cirrhosis; PBC, primary biliary cirrhosis.

Number	29
Age (mean (range))	66.0 (46–78)
Sex (male/female)	20/9
Underlying disease	
CLC	5
ALLC	4
NBNC	2
PBC	1
LC + HCC	17
Child-Pugh class	
A/B/C	14/14/1

**Table 3:** Sensitivity and specificity of ECE for diagnosing EV.

Detection of high-risk varices (F2 and/or RC2 ≦)	
Sensitivity	92% (=12/13)
Specificity	80% (=12/15)
Detection of RC sign	
Sensitivity	94% (=16/17)
Specificity	82% (=9/11)
Detection of esophageal varices	
Sensitivity	95% (=21/22)
Specificity	83% (=5/6)

## Abbreviations

ECE: Esophageal capsule endoscopy
EGD: Esophagogastroduodenoscopy
EV: Esophageal varices.
